# Longitudinal Analysis for Disease Progression via Simultaneous Multi-Relational Temporal-Fused Learning

**DOI:** 10.3389/fnagi.2017.00006

**Published:** 2017-03-03

**Authors:** Baiying Lei, Feng Jiang, Siping Chen, Dong Ni, Tianfu Wang

**Affiliations:** ^1^School of Biomedical Engineering, Shenzhen UniversityShenzhen, China; ^2^National-Regional Key Technology Engineering Laboratory for Medical Ultrasound, Shenzhen UniversityShenzhen, China; ^3^Guangdong Key Laboratory for Biomedical Measurements and Ultrasound Imaging, Shenzhen UniversityShenzhen, China; ^4^Fujian Provincial Key Laboratory of Information Processing and Intelligent Control, Minjiang UniversityFuzhou, China

**Keywords:** Alzheimer's disease (AD), longitudinal analysis, feature selection, joint learning, prediction

## Abstract

It is highly desirable to predict the progression of Alzheimer's disease (AD) of patients [e.g., to predict conversion of mild cognitive impairment (MCI) to AD], especially longitudinal prediction of AD is important for its early diagnosis. Currently, most existing methods predict different clinical scores using different models, or separately predict multiple scores at different future time points. Such approaches prevent coordinated learning of multiple predictions that can be used to jointly predict clinical scores at multiple future time points. In this paper, we propose a joint learning method for predicting clinical scores of patients using multiple longitudinal prediction models for various future time points. Three important relationships among training samples, features, and clinical scores are explored. The relationship among different longitudinal prediction models is captured using a common feature set among the multiple prediction models at different time points. Our experimental results based on the Alzheimer's disease neuroimaging initiative (ADNI) database shows that our method achieves considerable improvement over competing methods in predicting multiple clinical scores.

## Introduction

Alzheimer's disease (AD) imposes heavy social-economic burdens on society (Fan et al., 2008; Alzheimer's Association, 2014; Shi et al., 2015), and patients experience tremendous cognitive decline throughout progression of the AD disease. Tremendous effort have been devoted to improve the understanding and monitoring of AD progression (Brookmeyer et al., 2007; Hinrichs et al., 2009; Liu M. et al., 2014; Wang et al., 2014; Jie et al., 2015; Lei et al., 2015a; Liu et al., 2015; Lei et al., 2016; Zhuo et al., 2016). Modeling disease progression based on cognitive decline in longitudinal analysis has been widely investigated in the neuroimaging field (Fan et al., 2008; Davatzikos et al., 2010; Stonnington et al., 2010; Wang et al., 2010; Hinrichs et al., 2011). In recent decades, neuroimaging-based longitudinal studies have proven to be an important research direction in characterizing the neurodegenerative process of AD, where data at multiple time points are often used (Teipel et al., 2007; Vemuri et al., 2009; Jack et al., 2010; Cuingnet et al., 2011). It has been reported that researchers are able to study the cognitive decline due to the neurodegenerative property of AD with traditional structural magnetic resonance imaging (MRI) (Davatzikos et al., 2001, 2008; Dickerson et al., 2001; Gaser et al., 2001; Leow et al., 2006; Jack et al., 2008; Vemuri et al., 2008; Frisoni et al., 2010; Stonnington et al., 2010; Wang et al., 2016a), diffusion-weighted MRI (Jin et al., 2015, 2017; Daianu et al., 2016; Wang et al., 2016b; Wu et al., 2016), and functional MRI (Yang et al., 2016). In addition, cognitive decline in the neurodegenerative cognitive measures, e.g., the Alzheimer's disease assessment scale cognitive subscale (ADAS-Cog) and the mini mental state examination (MMSE), can be used to partially reveal AD progression (Davatzikos et al., 2001, 2008; Dickerson et al., 2001; Gaser et al., 2001; Leow et al., 2006; Jack et al., 2008; Vemuri et al., 2008; Frisoni et al., 2010). However, accurate prediction of AD progression still remains a challenging task due to the complicated characteristic of AD progression.

The first challenge in longitudinal studies for AD diagnosis is dimensionality of the data, which is usually much higher than the available number of samples. To address this issue, researchers have developed various feature selection models for different clinical scores (e.g., ADAS-Cog and MMSE) to identify disease-related biomarkers among multiple time points (Yuan and Lin, 2006; Zhang et al., 2012; Zhou et al., 2013). Among these methods, Lasso and its variants are the most popular techniques for feature selection (Tibshirani, 1996; Guyon et al., 2002; Guyon and Elisseeff, 2003; Yuan and Lin, 2006; Wang et al., 2010). For instance, (Wang et al., 2010), linear regression models are applied in high-dimensional pattern recognition problems not only to estimate the stage of AD, but also to construct a stable model. Adaptive regional feature extraction is applied to this model for the prediction of regression variables.

The second challenge is understanding the underlying relationship between features, subjects, and clinical scores at different time points. This relationship is seldom considered even though many longitudinal studies show promising predictive power in AD progression prediction. This relationship could provide inherent high-level information that is useful for studying AD. Therefore, modeling and utilizing this relationship could enhance the learning performance in predicting AD progression.

To tackle the above-mentioned challenges, several methods such as the group Lasso (Yuan and Lin, 2006), the temporally constrained group Lasso (TGL, Caroli and Frisoni, 2010; Jack et al., 2010), and the convex fused sparse group Lasso (cFSGL, Zhou et al., 2013) incorporated cognitive progression information into linear regression models to predict disease progression. Evidently, multi-task learning methods using intrinsic information achieve better performance than single-task learning methods (Liu et al., 2012, 2015; Zhu et al., 2014a; Jie et al., 2015), and this learning framework has shown great success in predicting ADAS-Cog and MMSE due to its good generalization capabilities (Zhang et al., 2011; Zhu et al., 2014a). For example, Zhang and Shen (Zhang et al., 2011) proposed a joint regression and classification scheme to understand the mechanism of AD. This method was further improved by Zhu et al. using a feature and variable graph matching method to jointly identify AD status and predict clinical scores (Zhu et al., 2014a). The inter-modality constraints described by Liu et al. were also included in a multi-task learning framework for AD diagnosis (Liu F. et al., 2014). Despite promising performance achieved by these methods, most existing methods fail to take advantage of the cognitive progression from multiple time points among features, subjects, and clinical scores. This mission is undesirable in longitudinal analysis and follow-up studies.

To incorporate cognitive progression relationships, longitudinal analysis has been widely explored to model cognitive progression and to exploit the associated imaging markers and cognitive changes across all time points (Misra et al., 2009; Davatzikos et al., 2010; Stonnington et al., 2010; Hinrichs et al., 2011; Zhang et al., 2012; Zhang and Shen, 2012; Zhou et al., 2013). For instance, Zhou et al. (2013) integrated temporal smoothness into their method using multi-task learning techniques to identify biomarkers for disease progression. Remarkable performance is achieved based on temporal-relational constraints and later-time constraints, in which each task is treated separately using a single baseline feature for predicting future-time-point score. Huang et al. (2015, 2016) proposed an improved random forest framework and took advantage of the longitudinal information at multiple time points to further improve the accuracy of AD score prediction. Using complementary information, such as MRI data and clinical scores, is desirable as it might uncover important imaging biomarkers. Zhang et al. found that longitudinal analysis is effective for mild cognitive interference (MCI) prediction (Zhang and Shen, 2012) and proposed the utilization of MRI features at multiple time points with temporal smoothness regularization (Zhang et al., 2012). This method outperformed competing methods, because the disease pattern is better revealed by comprehensive cognitive progression information as compared to methods using only the baseline features. In addition, the AD progression prediction problem in Wang et al. (2012) was addressed by a high-order multi-task learning method that exploits the temporal correlations in imaging and cognitive data with a structured sparsity inducing term. Promising predictive power is achieved via multiple time point features, and the clinical scores are learned independently. This arrangement is undesirable as correlation of the clinical score is ignored.

Most existing methods ignore the relationship among different features, subjects, and clinical scores. Furthermore, the relational information is seldom studied even though there is a strong correlation between the clinical scores and MRI data (Gaser et al., 2001; Leow et al., 2006). Moreover, most of the previous studies only focused on one or two types of relationships without considering the cognitive decline at different time points. The integration of this type of information would better identify the spatial patterns of brain atrophy because the associated feature patterns and the specific patterns of the neighboring time points are highly correlated. It has been also demonstrated in literature (Zhu et al., 2014a; Jie et al., 2015) that multiple relationships between feature-feature and subject-subject boost diagnosis performance.

Intuitively, information from clinical scores and subjects at multiple time-points can play an important role in identifying temporal patterns in longitudinal analysis. In this paper, a new multi-task joint feature learning method is developed to exploit the intrinsic relation of the data to boost performance of disease prediction. Manifold learning and discriminative learning theories have achieved remarkable performance by incorporating (Stanciu et al., 2014; Zhu et al., 2014b; Jie et al., 2015; Lei et al., 2015b; Zhou et al., 2015). Therefore, we explore temporal smoothness and multi-relation graphs among different patterns and cognitive measures to uncover human brain variations for better diagnosis of AD progression. Specifically, we define a novel objective function to impose multi-relation information. A group sparsity regularizer is used to jointly select a small number of specific features across different time points. We also incorporate multi-relation smoothness regularization to capture the relationship among features, subjects, and clinical scores. After the selection of longitudinal feature, the final selected brain regions are employed for clinical score prediction using multi-kernel support vector regression (SVR, Chang and Lin, 2011). To the best of our knowledge, there is no existing sparse model that incorporates multi-relation smoothness in its objective function to estimate the clinical scores (e.g., ADAS-Cog and MMSE). In addition, our method focuses on multiple relationships, which has obvious advantages over existing methods that only exploit feature or sample relationships. It is worth noting that we need to observe the behavioral changes in patients' condition over time in order to model disease progression. However, it is difficult to extract huge amount of information from data that is collected from multiple time points. Therefore, we utilize the aforementioned relational constraints to build a robust regression model by selecting the best and most relevant features to predict patient's clinical behavior at multiple future time points.

In this paper, we propose a joint learning procedure for multiple longitudinal predictions of AD progression by exploiting their inherent relationships. In particular, we propose three novel regularization terms (each modeling a set of crucial relationships at different time points), and incorporate these regularizers in a multi-task sparse feature selection model. We also introduce a specifically designed loss function to jointly predict the patients' clinical scores at multiple future time points, thus condensing the common information shared by data from different time points and permitting the selection of the most meaningful features for multiple prediction tasks. We evaluate our method using the Alzheimer's disease neuroimaging initiative (ADNI) (http://adni.loni.usc.edu/) database (Alzheimer's Association, 2014), and our method achieves promising results in estimating multiple clinical scores at multiple future time points using only baseline data. For the ADNI baseline, a total of 445 subjects—91 with AD, 202 with mild cognitive impairment (MCI), and 152 cognitively normal controls (NCs) are investigated in our study to predict the ADAS-Cog/MMSE scores for the next 2 years because the subjects already have completed MRI and clinical score data. In this work, we focus on only using suitable data instead of all the data from the ADNI database to study of disease progression.

Our extensive experimental results show that the proposed joint learning framework obtains state-of-the-art performance for future ADAS-Cog/MMSE score prediction. We observe that the hippocampal formation, amygdala, temporal pattern, and uncus demonstrate the most definitive patterns in predicting clinical scores at all-time points.

## Materials and methods

### Materials

Our investigation is based on data obtained from the ADNI database (Alzheimer's Association, 2014), which was created and updated since 2004. This 6-year study is funded $60 million from the public and private sectors, which include the National Institute on Aging, the National Institute of Biomedical Imaging, and Bioengineering, and the Food and Drug Administration. The principle goal of ADNI is to verify that serial MRI and positron emission tomography (PET) images, along with other biological markers, clinical, and neuropsychological assessment can be used to measure the progression of MCI and early AD. The determination of sensitive and specific markers of very early AD progression is intended to aid researchers and clinicians to develop new treatments and monitor their effectiveness, as well as to lessen the time and cost of clinical trials. ADNI is the collective effort of many co-investigators from a broad range of academic institutions and private corporations, and subjects have been recruited from over 50 sites across the US and Canada. 800 adults aged 55–90 were recruited to participate in this research, which includes approximately 200 cognitively normal older individuals followed for 3 years, 400 people with MCI followed for 3 years, and 200 people with early AD followed for 2 years. For up-to-date information, please refer to http://www.adni-info.org.

### Subjects and pre-processing

The general eligibility criteria of ADNI are briefly described in the following. Subjects between 55 and 90 years of age who have a study partner to provide an independent evaluation of functioning were selected. Patients taking certain psychoactive medications were excluded. There are three general inclusion/exclusion criteria: (1) The range of MMSE scores of healthy subjects (non-depressed, non-MCI, and non-demented) is 24–30; (2) The range of MMSE scores of MCI subjects is also between 24 and 30. The subjects having objective memory loss were measured with education adjusted scores using the Wechseler memory scale logical memory II, a CDR of 0.5, an absence of significant levels of impairment education adjusted scores fall between 20 and 26, and satisfy the National Institute of Neurological and Communicative Disorders and Stroke, and the Alzheimer's Disease and Related Disorders Association (NINCDS/ADRDA) criteria for probable AD. The study subjects gave written informed consent at the time of enrollment for imaging and genetic sample collection and completed questionnaires approved by each participating site Institutional Review Board (IRB). Table 1 gives the detailed ADAS-Cog and MMSE information of the subjects used in our study.

**Table 1:** **Statistical information of clinical scores**.

**Clinical scores**	**Mean**	**Median**	**Min**	**Max**
MMSE	12.02	10	0	59
ADAS-Cog	26.27	28	5	30

The pre-processing of feature extracted from the ROI regions have been widely applied in the literature (Liu et al., 2012, 2015; Zhu et al., 2014a; Jie et al., 2015). For our method, pre-processing was first applied to the T1-weighted MRI brain images of each subject, and then skull stripping was performed to clean the skull. The cerebellum was removed by warping a labeled Jacob atlas to the skull-stripped image (Wang et al., 2014). Segmentation by the FAST method (Zhang et al., 2001) was then applied to segment the brain images into three tissues, which include white matter (WM), gray matter (GM), and cerebrospinal fluid (CSF). After segmentation, the brain image was nonlinearly registered with a HAMMER tool (Shen and Davatzikos, 2002). The features used in this study include the volume intensity extracted from the region of interest (ROI) of different brain regions (Zhang et al., 2011). Specifically, the brain image of each subject was partitioned into 93 ROIs by atlas warping, and the volume of GM tissue of each ROI was extracted as a feature. Similar to the study in Wee et al. (2012), the obtained features were normalized to facilitate disease diagnosis and prognosis.

### Notation and problem statement

For this work, capital bold letters denote matrices, small bold letters denote vectors, and non-bold letters denote regular variables. Let X ∈ R^*S*×*F*^ denote the data of *S* different subjects, where each subject is represented by an *F*-dimensional feature vector from the baseline MRI image. Let X denote the data generated from baseline time point. We denote x_*u*,:_ and x_*v*,:_ as the *u*-the row vector and the *v*-th column vector of X, respectively. Let 𝕐 = {Y^(*t*)^ ∈ R^*S*×*C*^, *t* = 1, …, *T*} denote *C* types of clinical cognitive scores (e.g., ADAS-Cog and MMSE) for *S* subjects at *T* time points, where Y^(*t*)^ ∈ R^*S*×*C*^ is the corresponding clinical scores at the *t*-th time point for *S* subjects. Let 𝕎 = {W^(*t*)^ ∈ R^*F*×*C*^, *t* = 1, …, *T*} as denote the set of weight matrices that map the original features to clinical scores, where W^(*t*)^ represents the weight matrix for the *t*-th time point.

**Figure 1:**
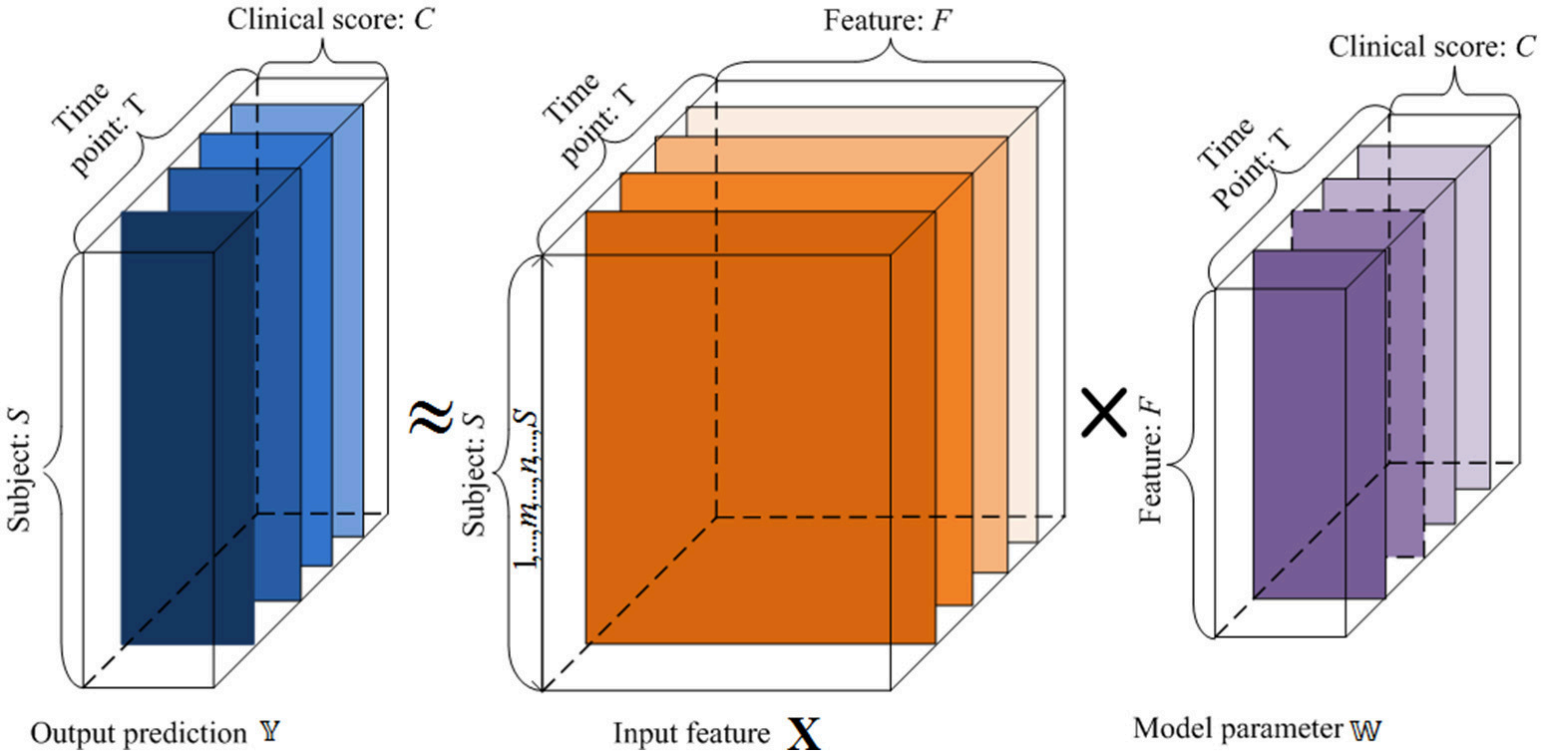
**An illustration of the proposed regression model using longitudinal data**. 𝕏 is the input baseline MRI data, 𝕐 is the target tensor data, and 𝕎 is the weight model projection. For the input 𝕏, *x*-axis represents the features, and *y*-axis denotes the subjects. For the target tensor data 𝕐, *x*-axis represents clinical scores, *y*-axis represents the subjects, and *z*-axis denotes the time points. For the model parameters 𝕎, *x*-axis plots the clinical scores, *y*-axis represents the features, and *z*-axis denotes the time points. We want to establish a linear model 𝕎 between the input 𝕏 and output 𝕐.

Our goal is to create a linear regression model to reveal the longitudinal associations between the original features and the cognitive trajectories through time, and predict the clinical scores at multiple future time points from the baseline data (*t* = 1). This is illustrated in Figure 1. Each subject's features are assigned as a row in the matrix X. By learning the weight vectors in each W^(*t*)^, we can reconstruct the corresponding clinical scores in each Y^(*t*)^, as explained below.

**Figure 2:**
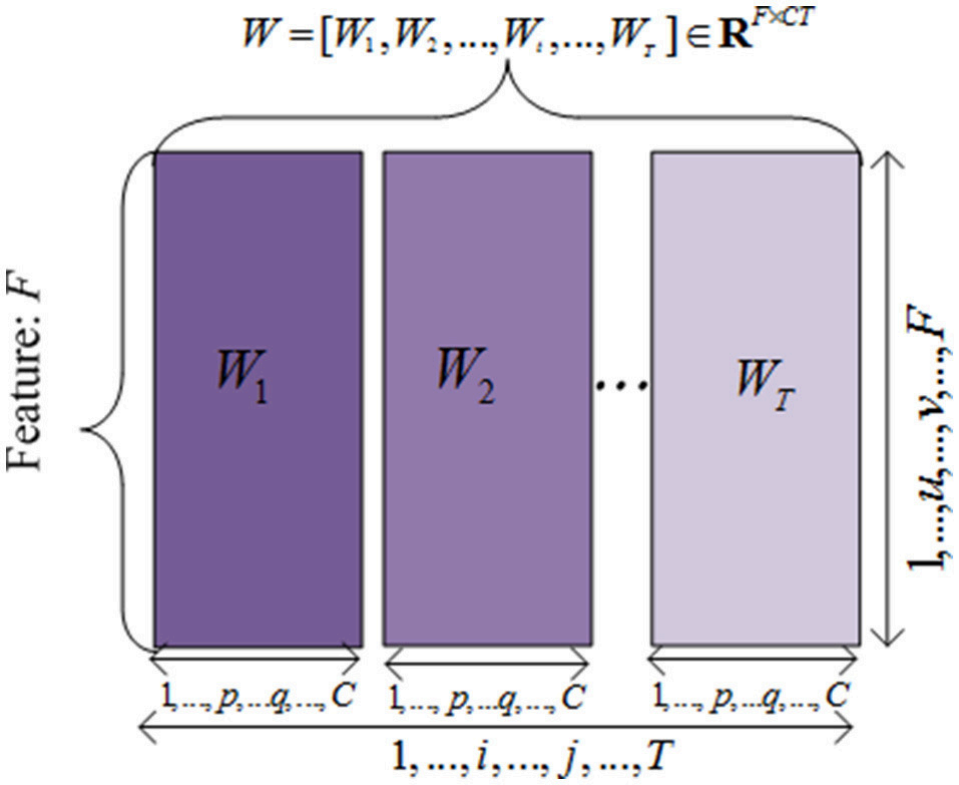
**The illustration of the unfolded weight matrices**. Each row is correponding to a specific feature vector among different time points, and each column is corresponding to a clinical score vector at one time point.

To simplify the problem and design the objective function, we unfolded the projected weight matrix by concatenating all W^(*t*)^ s as $\hat{\text{W}}=\left[{\text{W}}^{\left(1\right)},{\text{W}}^{\left(2\right)},\cdots \;,{\text{W}}^{\left(t\right)},\cdots \;,{\text{W}}^{\left(T\right)}\right]\in {\text{R}}^{F\times CT}$. Hence, the model parameters can be incorporated in the objective function to extract the common features across different time points. This unfolded simplified weighting matrix is shown in Figure 2.

### Methodology

**Figure 3:**
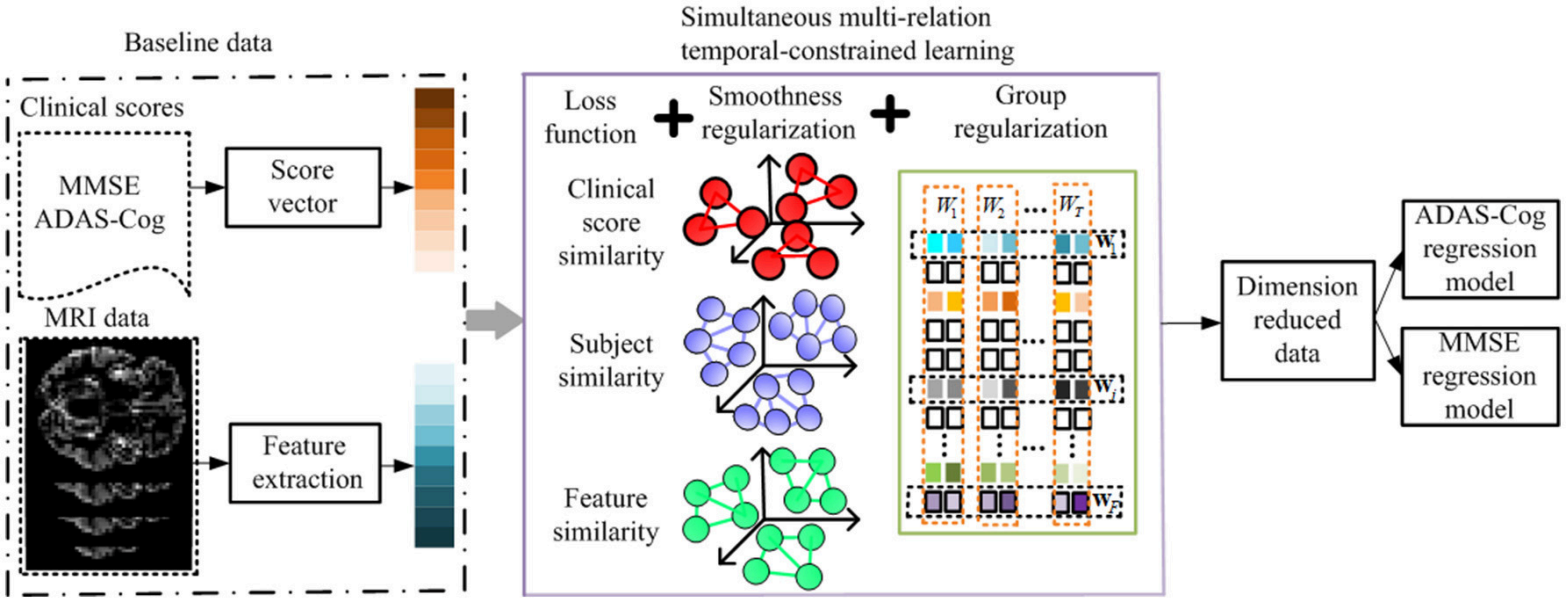
**Flowchart of the proposed method**. The MRI data and clinical scores are extracted for longitudinal feature selection, with smoothness regularization (i.e., feature-feature, subject-subject, and clinical score-clinical score relation guided regularizers) and a group sparsity induced regularization. After longitudinal feature selection, the feature dimension is reduced, and the selected features are employed to build ADAS-Cog and MMSE regression models for prediction.

A key advantage of longitudinal studies is the ability to observe the patients' changes through time, and to effectively utilize the shared common information in different time points to select the best set of features for monitoring the progression of MCI patients and thereafter predicts their future status. There are three different aspects in which this common information could be leveraged, i.e., the relationships among features, subjects, and clinical scores. Intuitively, the pairwise similarities among features, subjects, and clinical scores should be preserved in the predictions via the regression model. In this section, we introduce a method to incorporate such relation information into a multi-task learning framework. Specifically, we define a linear regression model for each time point using the baseline data as a single task, and then formulate the global regression model in a multi-task learning framework with a *l*_2, 1_ sparsity constraint, where the above three relational aspects are incorporated as regularization terms. Figure 3 shows the flowchart of the proposed method.

**Figure 4:**
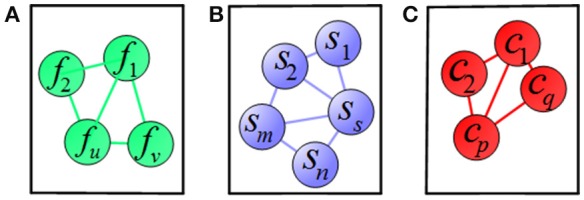
**Illustration of relations among (A)** features, **(B)** subjects, and **(C)** clinical scores, where a node means a vector of **(A)** feature, **(B)** subject, **(C)** clinical score, respectively, and an edge denotes the distance between the nodes.

To clearly illustrate the relationship among features, subjects, and clinical scores, we adopted a graph matching technique in our proposed method, where the feature-feature, subject-subject and clinical score-clinical score relationships are represented in terms of graph (shown in Figure 4). In Figure 4A, a node represents one feature and an edge represents the relationship between the connected nodes. Figures 4B,C show the relationship among subjects and among clinical scores, respectively. In these graphs, the length of an edge between features (subjects or clinical scores) represents the similarity among features (subjects or clinical scores), where the similarity increases with the length of the edge. These graphs are built based on the information of training data, which are then used as regularization terms for subsequent multi-task learning procedure.

Previous studies (e.g., Zhou et al., 2013) revealed the associations among imaging features and cognitive scores at each time point separately, under the assumption that each task at each time point is independent. However, this assumption does not always hold because clinical scores possess temporal correlation. In fact, harnessing the temporal correlation could potentially help predict the clinical cognitive scores. This motivates us to use a joint learning regression model across all time points, which would help identify the most relevant imaging markers for the prediction of cognitive scores. Specifically, we seek to learn the weight coefficient matrices to uncover the clinical scores progression, through which the information from each learning task and the common structures among multiple time points can be jointly discovered.

To select the most relevant and discriminant features at each time point, a correlation-induced sparsity model with a least-squares loss function is proposed. The loss function controls the prediction error, while the sparsity assumption leads to the least number of contributing features. As a result, for each time point, we would select the features that are most correlated with the actual clinical scores. A general form of the proposed objective function is defined as below:

(1) $$\begin{array}{l} \underset{\left\{W^{\left(t\right)},t\;=\;1,..,T\right\}}{\min}\sum_{t\;=\;1}^{T}∥Y^{\left(t\right)}-XW^{\left(t\right)}{∥}_{F}^{2}\\ +\;\lambda_{1}\sum_{t\;=\;1}^{T}\Phi \text{​}\left(W^{\left(t\right)}\right)+\lambda_{2}∥\hat{W}{∥}_{2,1}, \end{array}$$

where $∥\cdot {∥}_{F}^{2}$ is the Frobenius norm of a matrix, **W** is the coefficient weight matrix, λ_1_ and λ_2_ are the regularization parameters. The last term in Equation (1) is a group regularizer to uncover the correlation among different features and jointly select features for multiple tasks, which is defined as $∥\hat{\text{W}}{∥}_{2,1}=\sum_{i\;=\;1}^{F}∥{\hat{w}}_{i,:}{∥}_{2}$, where ${\hat{w}}_{i,:}$ is the *i*-th row vector of $\hat{\text{W}}$, and $∥\cdot {∥}_{F}^{2}$ is *l*_2, 1_-norm. It is worth noting that *l*_2, 1_ -norm computes the sum of the *l*_2,1_ -norm of each row of $\hat{\text{W}}$, which enforces many rows to be zero, and hence it is suitable for feature selection. Features corresponding to those non-zero rows in $\hat{\text{W}}$ are regarded as the most predictive features in subsequent learning models. The second term Φ(**W**^(*t*)^) is our proposed regularizer, which is comprised of multiple parts corresponding to three relationships among features, among subjects, and among clinical scores. To define the proposed regularization term, Φ(**W**^(*t*)^), the “feature-feature,” the “subject-subject,” and the “clinical score-clinical score” relationships at each different time point are incorporated. We use the idea of Laplacian matrices and graphs to obtain the similarity in the local structures (Belkin and Niyogi, 2003). In the rest of this section, we explain it in detail and discuss all its characteristics.

The “feature-feature” relation information is imposed as the relationship between columns of the input matrix X, and is reflected in the relation between corresponding rows in the coefficients weight matrix W^(*t*)^. Hence, the widely used graph Laplacian (Hinrichs et al., 2009; Wang et al., 2012; Zhu et al., 2013) is leveraged. To measure the similarity between the *u*-th and the *v*-th features of X. in the original feature space, we use the heat kernel defined as below:

(2) $$f_{uv}=\exp\left(-∥x_{:,u}-x_{:,v}{∥}_{2}^{2}\right),$$

where **x**_:,*u*_ is the *u*-th column of the input data **X**. Based on the similarity, we develop the first feature-feature relation based regularization term as

(3) $$R_{f}\text{​}\left(W^{\left(t\right)}\right)=\sum_{u,v\;=\;1}^{F}f_{uv}∥w_{u,:}^{\left(t\right)}-w_{v,:}^{\left(t\right)}\text{​}{∥}_{2}^{2},$$

where ${\text{w}}_{u,:}^{\left(t\right)}\;$ is the *u*-th row of **W**^(*t*)^ at time point *t*. Thus, the highly correlated features produce large weights in the above sparsity regularization.

The second regularization is based on the “subject-subject” relation graph. We know that the output clinical scores of similar subjects should be similar. Therefore, similar to the previous term, we use a heat kernel to exploit the “subject-subject” similarities and define the similarity between the *m*-th and the *n*-th subject as

(4) $$\phi_{mn}=\exp\left(-∥x_{m,:}-x_{n,:}{∥}_{2}^{2}\right),$$

where **x**_*m*,:_ is the *m*-th row of input **X**. Here, “subject-subject” relation regularizer is defined as

(5) $$R_{s}\text{​}\left(W^{\left(t\right)}\right)=\sum_{m,n\;=\;1}^{S}\phi_{mn}∥\text{​}x_{m,:}W^{\left(t\right)}-x_{n,:}W^{\left(t\right)}{∥}_{2}^{2}.$$

The last regularization is based on the “clinical score-clinical score” relation. For each subject's feature vector x_*p*,:_ in our regression framework, different sets of weight coefficients are used to regress the output clinical scores ${\text{y}}_{:,p}^{\left(t\right)}$. In other words, the elements in each column of W^(*t*)^ are related to the elements in each column of Y^(*t*)^ through the feature vectors. As a result, if two clinical scores are correlated, the corresponding weight columns in matrix W^(*t*)^ should be correlated too. Similarly, we use a heat kernel to exploit the “clinical score-clinical score” relation. The similarity between the *p*-th clinical score and the *q*-th clinical score is defined using a heat kernel as

(6) $$\psi_{pqt}=\exp\left(-∥\text{​ }y_{:,p}^{\left(t\right)}-y_{:,q}^{\left(t\right)}{∥}_{2}^{2}\right),$$

where ${\text{y}}_{:,p}^{\left(t\right)}$ is the *p*-th column vector of **Y**^(*t*)^. To this end, we define the clinical score relational regularization term as

(7) $$R_{c}\text{​}\left(W^{\left(t\right)}\right)=\sum_{p,q\;=\;1}^{C}\text{​}\text{​}\psi_{pqt}∥w_{:,p}^{\left(t\right)}-w_{:,q}^{\left(t\right)}{∥}_{2}^{2}.$$

Therefore, our proposed joint learning model using the relation information, as discussed above, is

(8) $$\begin{array}{l} \underset{\left\{W^{\left(t\right)},t\;=\;1,..,T\right\}}{\text{min}}\sum_{t\;=\;1}^{T}\text{​}∥Y^{\left(t\right)}-XW^{\left(t\right)}{∥}_{F}^{2}+\;\lambda_{1}\sum_{t\;=\;1}^{T}\text{​}(R_{f}\left(W^{\left(t\right)}\right)\\ +\;R_{s}\text{​}\left(W^{\left(t\right)}\right)+R_{c}\text{​}\left(W^{\left(t\right)}\right))+\;\lambda_{2}∥\text{​​}\hat{W}{∥}_{2,1}\text{​}. \end{array}$$

Using Equation (8), we incorporate three types of relationship data at multiple time points into a unified objective function. This method is referred as the simultaneous multi-relation temporally constrained learning (SMTL). To the best of our knowledge, this is the first work to simultaneously incorporate multi-relation information such as “feature-feature,” “subject-subject,” and “clinical score-clinical score” in fused regularizations, which is difficult to solve in current sparse models. In addition, no previous studies jointly apply the multi-relation information across multiple time points as additional regularizers.

Motivated by Zhu et al. (2013), the optimization problem in Equation (8) could be solved in an alternative way (i.e., finding the optimal solution for one variable while the others are fixed). The optimization steps are discussed in the following section. After selecting the most meaningful features, we use a SVR model to predict the clinical scores of patients at multiple future time points. Considering how AD changes over time, our algorithm benefits from a joint multi-task learning framework, in which multiple relationships are introduced as regularization terms to take advantage of the local structure similarity in the data. Because of the sparsity property of the *l*_2,1_ -norm regularization on weight vectors, the optimal weights contain some zero or close to zero row vectors. Structured sparsity is then imposed through penalizing all the regression coefficients corresponding to each single feature, at multiple time points. Thus, the most distinctive and predictive features will have similar large weights across all time points.

### Optimization algorithms

Although our objective function is convex, it is difficult to solve because regularization terms are based on non-smooth sparsity-inducing norms in the objective function (Zhu et al., 2013, 2014a). l_2, 1_ norm minimization is more challenging to solve than the l_1_ -norm minimization problem. As most existing optimization algorithms are too computation costly to solve our problem (Wee et al., 2012; Zhang and Shen, 2012), an efficient iterative algorithm is developed in this work.

In the similarity measurement, a Laplacian graph at each time point is built based on a diagonal matrix and formulated as: $D_{f}=f_{uv},D_{s}=\phi_{mn},D_{c}^{\left(t\right)}=\psi_{pqt}$ Let *S_f_*, *S_s_* and $S_{c}^{\left(t\right)}$ denote the summation of the diagonal entry of *D_f_*, *D_s_*, and $D_{c}^{\left(t\right)}$, respectively. The graph Laplacian *L_f_* for the feature space at each time point is: *L_f_* = *D_f_* − *S_f_*. Similarly, we have the Laplacian graph for the subject and clinical score, *L_s_* = *D_s_* − *S_s_* and $L_{c}^{\left(t\right)}$ = $D_{c}^{\left(t\right)}$ − $S_{c}^{\left(t\right)}$, respectively. As any regularization term $R_{s}\left({\text{W}}^{\left(t\right)}\right)$ can be reformulated as $\;R_{f}({\text{W}}^{\left(t\right)})=Tr\left({\left({\text{W}}^{\left(t\right)}\right)}^{T}L_{f}{\text{W}}^{\left(t\right)}\right)\text{​},R_{s}({\text{W}}^{\left(t\right)})=Tr\left({\left({\text{W}}^{\left(t\right)}\right)}^{T}L_{s}{\text{W}}^{\left(t\right)}\right)\text{​},R_{c}({\text{W}}^{\left(t\right)})=Tr\left({\left({\text{W}}^{\left(t\right)}\right)}^{T}L_{c}^{\left(t\right)}{\text{W}}^{\left(t\right)}\right)$. Assuming *L*_*D*_ is Laplacian graph built based on a diagonal matrix of $\hat{\text{W}}$, the objective is first reformulated as:

(9) $$\begin{array}{l} J\left(W^{\left(\text{t}\right)}\right)=\underset{\left\{W^{\left(t\right)},t\;=\;1,..,T\right\}}{\text{min}}\sum_{t\;=\;1}^{T}∥Y^{\left(t\right)}-XW^{\left(t\right)}{∥}_{F}^{2}\\ +\;\lambda_{1}\sum_{t\;=\;1}^{T}Tr({\left(W^{\left(t\right)}\right)}^{T}L_{f}\;W^{\left(t\right)}+{\left(XW^{\left(t\right)}\right)}^{T}L_{s}\;XW^{\left(t\right)}\\ +\;W^{\left(t\right)}L_{\text{c}}^{\left(t\right)}{\left(W^{\left(t\right)}\right)}^{T})+\lambda_{2}Tr\text{​}\left({\hat{W}}^{T}L_{D}\hat{W}\right), \end{array}$$

where *L*_*D*_ is Laplacian graph built based on a diagonal matrix of $\hat{\text{W}}$. The optimal solution of W^(*t*)^ is obtained by taking the derivative of the objective function with respect to W^(*t*)^.

By taking the derivative of the objective function in Equation (8) with respect to W^(*t*)^ and set to 0, we obtain:

(10) $$\begin{array}{l} X^{T}X-X{\left(Y^{\left(t\right)}\right)}^{T}+\;\lambda_{1}\left(L_{f}\;W^{\left(t\right)}+X^{T}L_{s}\;XW^{\left(t\right)}+W^{\left(t\right)}L_{\text{c}}^{\left(t\right)}\right)\\ \;+\;\lambda_{2}L_{D}\;W^{\left(t\right)}=0. \end{array}$$

We can rewrite Equation (10) as

(11) $$\begin{array}{l} \left(X^{T}X+\lambda_{1}L_{f}+\lambda_{1}X^{T}L_{s}\;X+\lambda_{2}L_{D}\right)W^{\left(t\right)}+W^{\left(t\right)}\left(\lambda_{1}L_{\text{c}}^{\left(t\right)}\right)\\ =X{\left(Y^{\left(t\right)}\right)}^{T}. \end{array}$$

This equation is regarded as Sylvester equation and solvable in the closed form Zhu et al. (2014a) using

(12) $$AW^{\left(t\right)}+W^{\left(t\right)}B=Q,$$

where $A=X^{T}X+\lambda_{1}L_{f}+\lambda_{1}X^{T}L_{s}\;X+\lambda_{2}L_{D},B=\lambda_{1}L_{\text{c}}^{\left(t\right)}$, $\text{B}=\lambda_{1}{\text{L}}_{\text{c}}^{\left(t\right)}$ and **Q** is **X**(**Y**^(*t*)^)^***T***^, **W**^(*t*)^, (1 ≤ *t* ≤ *T*) can be obtained by solving the Sylvester equations (Zhu et al., 2014a) when the time *t* changes from 1 to *T*.

Since *L*_*f*_, *L*_*s*_, $L_{c}^{\left(t\right)}$, and *L*_*D*_ are obtained from W^(*t*)^ and are dependent of W^(*t*)^, an iterative optimization is proposed to efficiently obtain the global solutions of W^(*t*)^, (1 ≤ *t* ≤ *T*) (Zhu et al., 2013). The solution of W^(*t*)^ for1 ≤ *t* ≤ *T* is summarized in Algorithm 1. The iterative optimization method updates W^(*t*)^ until the objective function converged.

**Algorithm 1:** **An iterative algorithm to solve the optimization problem in Equation (8)**.

Input:	Baseline MRI training data of *S* subjects and *F* dimensional feature: **X** ∈ **R**^*S*×*F*^ *T* time points clinical scores of *S* subjects and *C* dimensional clincial score vector: 𝕐 = {**Y**^(*t*)^ ∈ **R**^*S*×*C*^, *t* = 1, …, *T*} Parameters: regularization paramters and iteration times
Output:	Weight projection matrix: 𝕎 = {**W**^(*t*)^ ∈ **R**^*F*×*C*^, *t* = 1, …, *T*}
	Set iteration *r* = 0 and initialize **W**^(*t*)^ ∈ **R**^*F*×*C*^ according to the linear model for each time point;
	Initialization: ${\hat{\text{W}}}^{\left(0\right)}=\left[{\text{W}}^{\left(1\right)},{\text{W}}^{\left(2\right)},\ldots ,{\text{W}}^{\left(t\right)},\ldots ,{\text{W}}^{\left(T\right)}\right]$
	**Repeat**
	**for** *t* = 1 **to** *T*
	Calculate ***L***_***f***_, ***L***_***s***_, $L_{c}^{\left(t\right)}$ and ***L***_***D***_, according to the above definitions;
	Update ${\hat{\text{W}}}_{r}^{\left(t\right)}$ by solving the Sylvester problem in equation (13);
	**End for**
	${\hat{\text{W}}}_{r+1}^{\left(t\right)}=\left[{\text{W}}^{\left(1\right)},{\text{W}}^{\left(2\right)},\ldots ,{\text{W}}^{\left(t\right)},\ldots ,{\text{W}}^{\left(T\right)}\right]$;
	*r* = *r* + 1;
	**until** (*r* = 50 or $\hat{\text{W}}-{\text{W}}_{\text{2}}<10^{–6}$)
Return	**W**^(*t*)^, (1 ≤ *t* ≤ *T*)

## Experimental results

A 10-fold cross validation strategy is employed to avoid any bias introduced in the data and experiments. A set of 445 subjects comprising of 91 NC, 202 patients with MCI, and 152 AD patients are included in our study. We also used subjects from the ANDI database with T1-weighted MRI data from a 1.5 T scanner. The entire set of subjects is equally partitioned into 10 subsets, and the subjects of one subset are selected as the testing samples and the subjects in the remaining nine subsets are used for training regression models. In our experiments, the regression model is implemented using the LIBSVM toolbox with default parameters, and a linear kernel is adopted after normalizing each feature vector into a unit norm. In the pre-processing step, the features are z-normalized by removing their mean and dividing the result by its standard deviation. The two cognitive measurements, ADAS-Cog and MMSE, are computed from the MRI data collected at four different time points. The experimental setup from Zhou et al. (2013) is adopted. The regularization parameters in the feature-selection model of Equation (8) (i.e., λ_1_ and λ_2_) are determined by performing another round of cross-validation on the training data.

**Figure 5:**
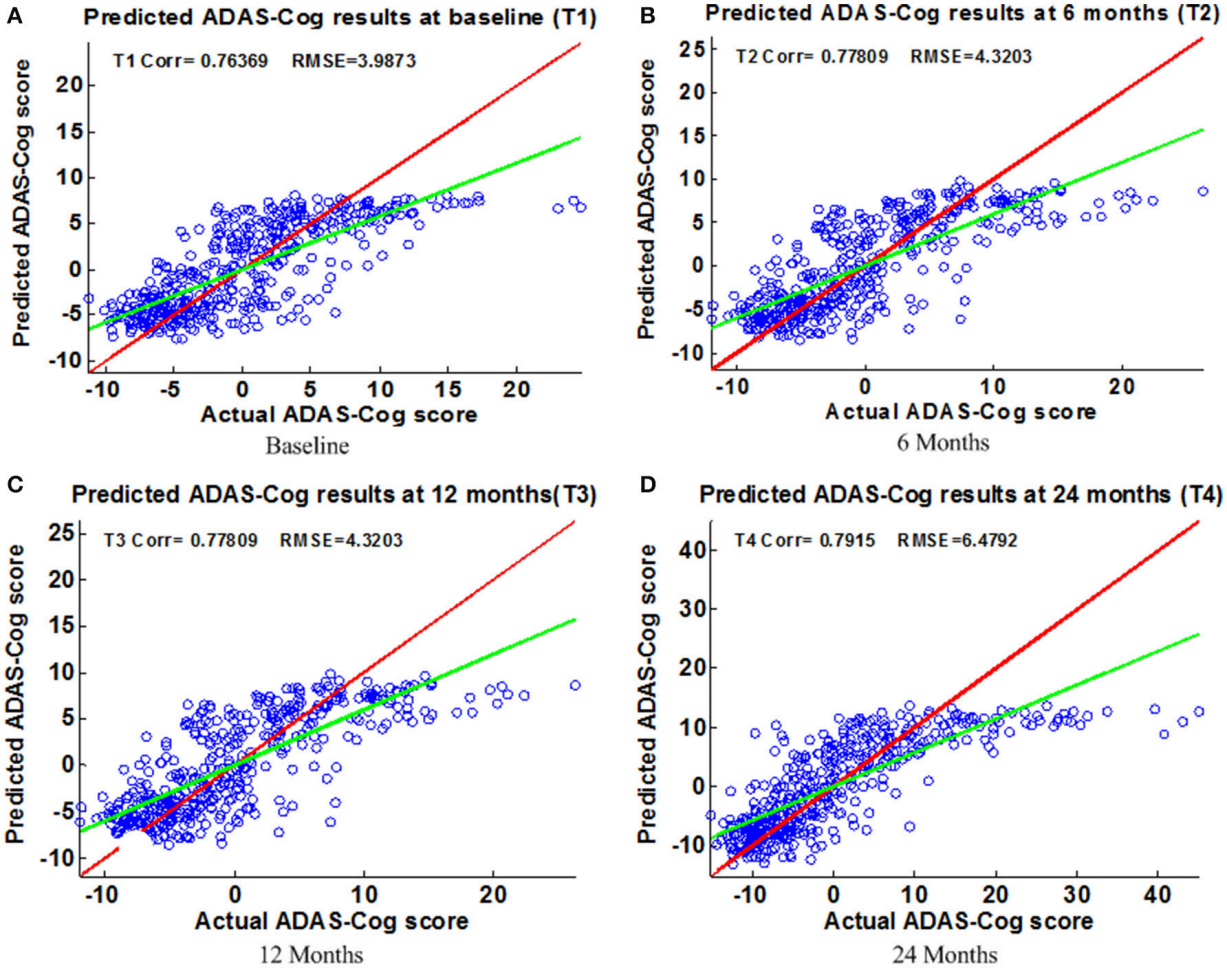
**Scatter plots of predicted vs. actual ADAS-Cog values at different time points; (A)** Baseline **(B)** 6 Months **(C)** 12 Months **(D)** 24 Months. The red line in each figure is a reference of perfect correlation. The green line is the regression line by the proposed model. The closer between the regression line and reference line, the better performance the proposed method can achieve. A high correlation is observed for ADAS-Cog prediction at each time point.

Figure 5 summarizes the objective function values at different iterations. The objective function values monotonically decrease as the number of iterations increases, which is consistent with our convergence analysis. The objective value quickly converged after a few iterations, which demonstrates the effectiveness of the proposed optimization method and the efficacy of our feature selection algorithms.

Extensive experiments are performed to evaluate the effectiveness of the proposed feature selection method. Two regression tasks are constructed to predict the changes in ADAS-Cog and MMSE for the baseline and a two-year follow-up study. The widely used Pearson's correlation coefficient (Corr) and root-mean-square error (RMSE) metrics are used to measure performance (Duchesne et al., 2009; Ito et al., 2010; Stonnington et al., 2010; Zhou et al., 2013).

### ADAS-cog and MMSE prediction results

**Figure 6:**
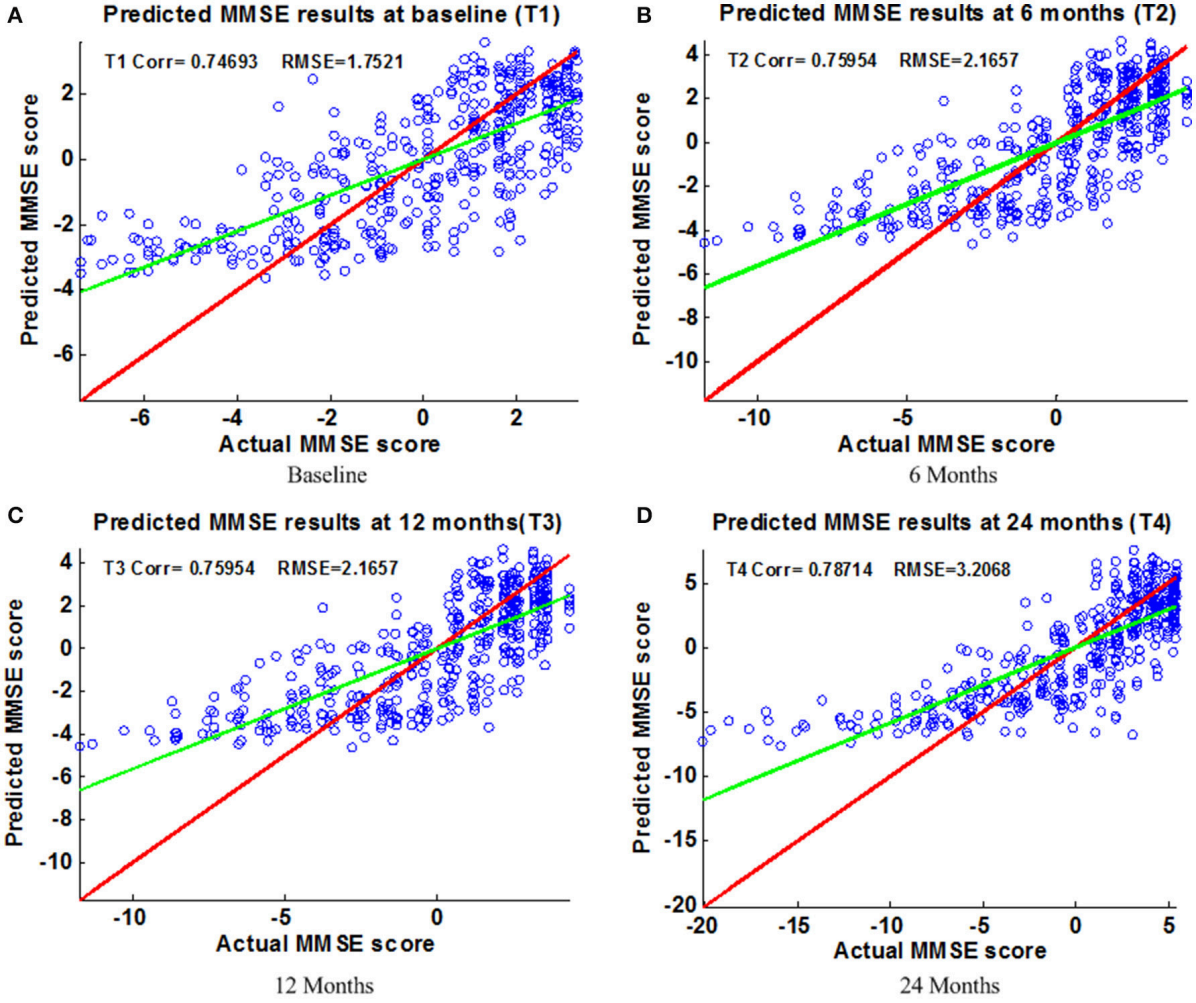
**Scatter plots of predicted vs. actual MMSE values at different time points; (A)** Baseline **(B)** 6 Months **(C)** 12 Months **(D)** 24 Months. The red line in each figure is a reference of perfect correlation. The green line is the regression line with the proposed model. The closer between the regression line and reference line, the better prediction the proposed model can achieve. A high correlation is observed for MMSE prediction at each time point.

**Figure 7:**
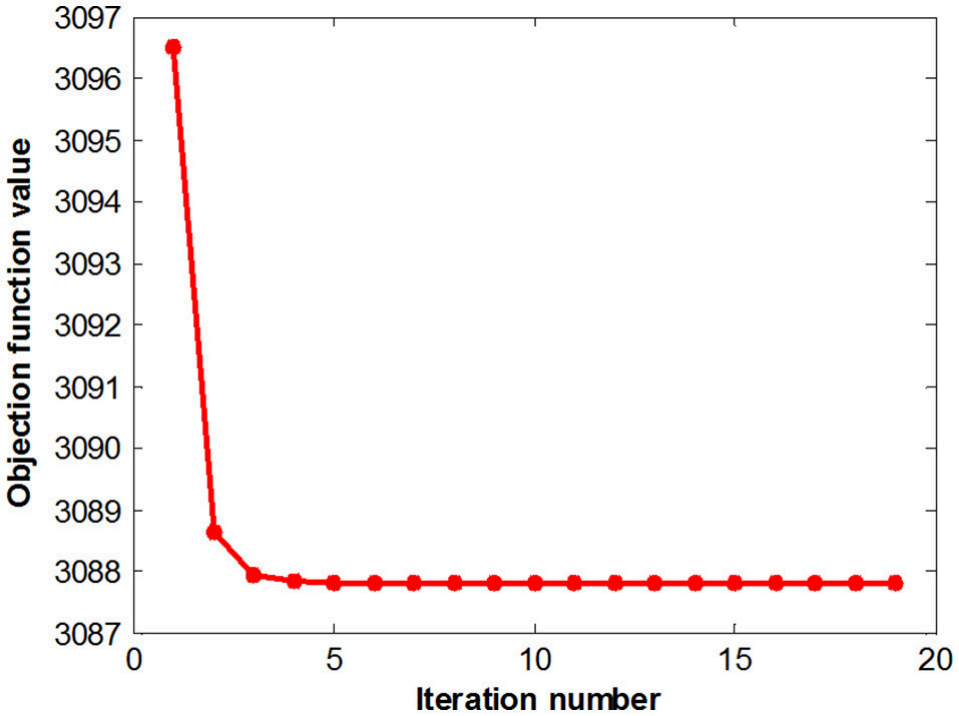
**Objective value as a function of the iteration number on the ADNI dataset**.

Figure 6 shows the scatter plots of the estimated ADAS-Cog scores vs. the actual ADAS-Cog scores obtained with the proposed method at baseline, 6, 12, and 24 months. Figure 7 shows the scatter plots of the predicted MMSE vs. the actual MMSE scores obtained with the proposed method at baseline, 6, 12, and 24 months. The linear model adopted in the proposed method is illustrated with a red line, and the perfect regression method is shown by a green line for comparison. Although predicting future clinical scores is quite challenging, it is evident that our proposed method achieves remarkable results in terms of Corr and RMSE results. Similar to the previous studies (Zhang and Shen, 2012; Zhang et al., 2012; Zhou et al., 2013), we observed that predicting early and changes of MCI up to 1 year is more difficult than later time points as less distinct information is available to separate early MCI. The low correlation values in the early time periods are mainly due to the failure to detect progression from MCI to early AD, and it is even harder to uncover essential changes of brain regions in early MCI. Our experimental results show that the proposed multiple time point joint learning with multiple relationships information is better at predicting ADAS-Cog and MMSE scores than single-task learning and the separate learning methods. We also address the problem of predicting the future cognitive decline of MCI subjects.

### Algorithm comparison

**Figure 8:**
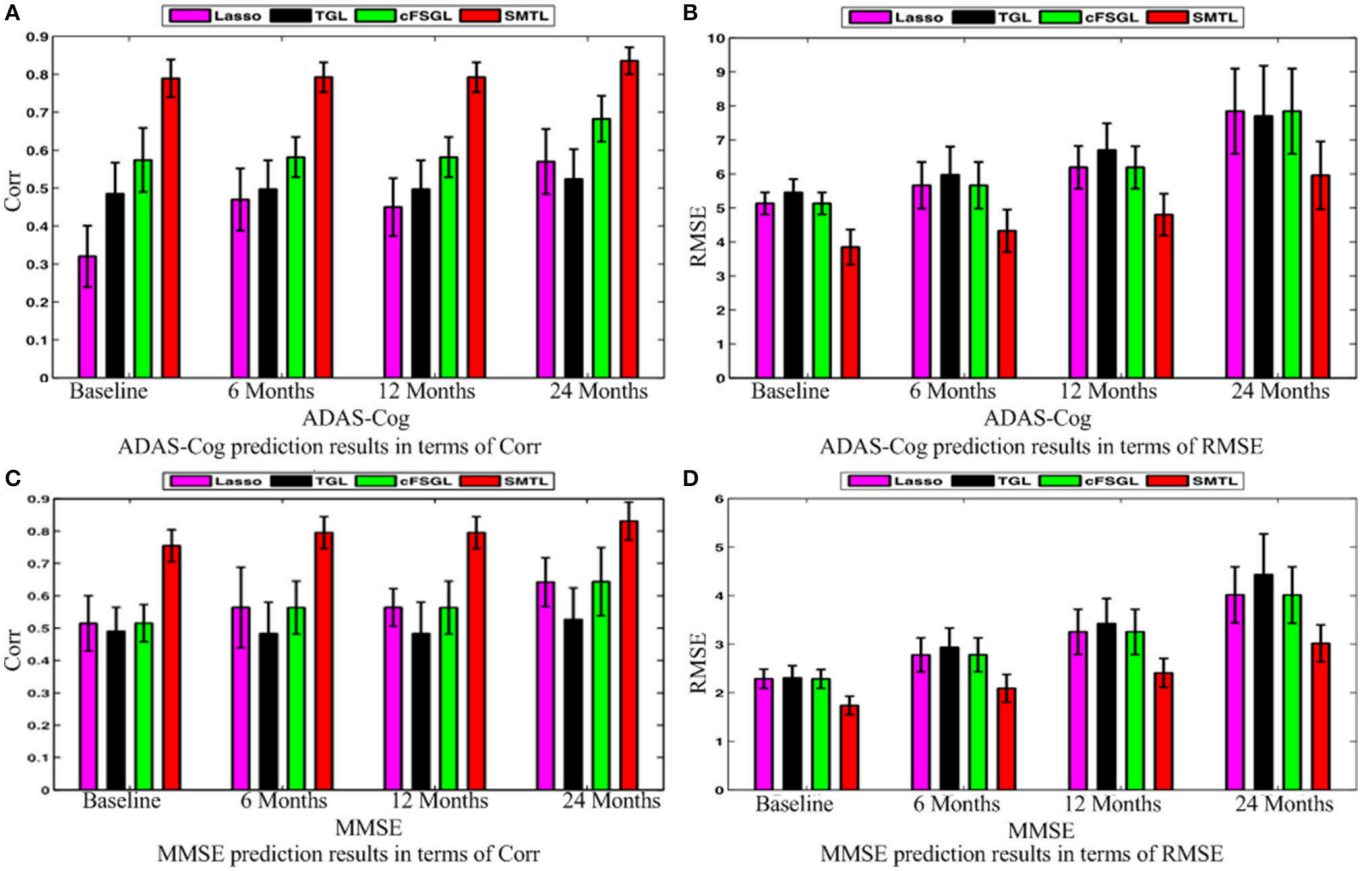
**Comparison of ADAS-Cog and MMSE prediction results by our proposed SMTL method and three feature selection methods: Lasso, TGL, and cFSGL, using Corr and RMSE measurement; (A)** ADAS-Cog prediction results in terms of Corr **(B)** ADAS-Cog prediction results in terms of RMSE **(C)** MMSE prediction results in terms of Corr. **(D)** MMSE prediction results in terms of RMSE.

A comparison of our proposed method with three feature selection methods, namely, Lasso, temporal group Lasso (TGL, Zhang et al., 2012), and convex fused temporally constrained group Lasso (cFSGL, Zhou et al., 2013) is performed. Figure 8 shows the performance of these methods on predicting ADAS-Cog/MMSE scores at baseline (T1), 6 months (T2), 12 months (T3), and 24 months (T4). We also present the detailed algorithm comparison results in terms of Corr and RMSE in Table 2. Our experimental results demonstrate that the proposed method performs better than the separate learning method in predicting ADAS-Cog and MMSE scores. We observed that the prediction of the changes of early and first year clinical score is significantly harder than the later time points since there is less distinct information available for the earlier prediction, which was also confirmed by previous studies (Zhang et al., 2012; Zhou et al., 2013). The main reason for low correlation in the early time frame is that there is not sufficient time for MCI to progress to early AD, thus it is more challenging to uncover the essential changes of brain regions in early MCI. Our proposed method achieves stable and promising results for both ADAS-Cog and MMSE prediction, and outperforms several state-of-the-art methods (Zhang et al., 2012; Zhou et al., 2013). From the experimental results, the promising prediction results clearly suggest the effectiveness of the proposed method.

**Table 2:** **Comparison of ADAS-Cog and MMSE prediction results by our proposed SMTL method and three feature selection methods: Lasso, TGL, and cFSGL, using Corr and RMSE measurement**.

	**ADAS–Cog**				**MMSE**			
	**Lasso**	**TGL**	**cFSGL**	**SMTL**	**Lasso**	**TGL**	**cFSGL**	**SMTL**
Baseline Corr	0.32 ± 0.08	0.49 ± 0.08	0.58 ± 0.08	0.77 ± 0.05	0.51 ± 0.09	0.56 ± 0.12	0.64 ± 0.08	0.75 ± 0.08
M06 Corr	0.48 ± 0.07	0.50 ± 0.07	0.59 ± 0.05	0.78 ± 0.04	0.49 ± 0.07	0.48 ± 0.10	0.63 ± 0.10	0.79 ± 0.10
M12 Corr	0.46 ± 0.12	0.52 ± 0.09	0.62 ± 0.05	0.79 ± 0.04	0.52 ± 0.06	0.57 ± 0.08	0.64 ± 0.12	0.79 ± 0.12
M24 Corr	0.57 ± 0.08	0.53 ± 0.08	0.69 ± 0.05	0.84 ± 0.04	0.61 ± 0.05	0.58 ± 0.05	0.65 ± 0.05	0.83 ± 0.06
Baseline RMSE	5.19 ± 0.33	5.53 ± 0.32	5.20 ± 0.33	3.81 ± 0.45	2.25 ± 0.20	2.30 ± 0.24	2.24 ± 0.21	1.75 ± 0.20
M06 RMSE	5.48 ± 0.62	5.90 ± 0.65	5.48 ± 0.63	4.36 ± 0.46	2.75 ± 0.31	2.85 ± 0.33	2.76 ± 0.32	2.31 ± 0.29
M12 RMSE	6.25 ± 0.58	6.74 ± 0.63	6.24 ± 0.59	4.91 ± 0.56	3.42 ± 0.49	3.59 ± 0.52	3.42 ± 0.50	2.48 ± 0.40
M24 RMSE	7.95 ± 1.20	7.81 ± 1.39	7.96 ± 1.23	6.00 ± 1.00	4.05 ± 0.58	4.48 ± 0.82	4.04 ± 0.58	3.00 ± 0.38

**Figure 9:**
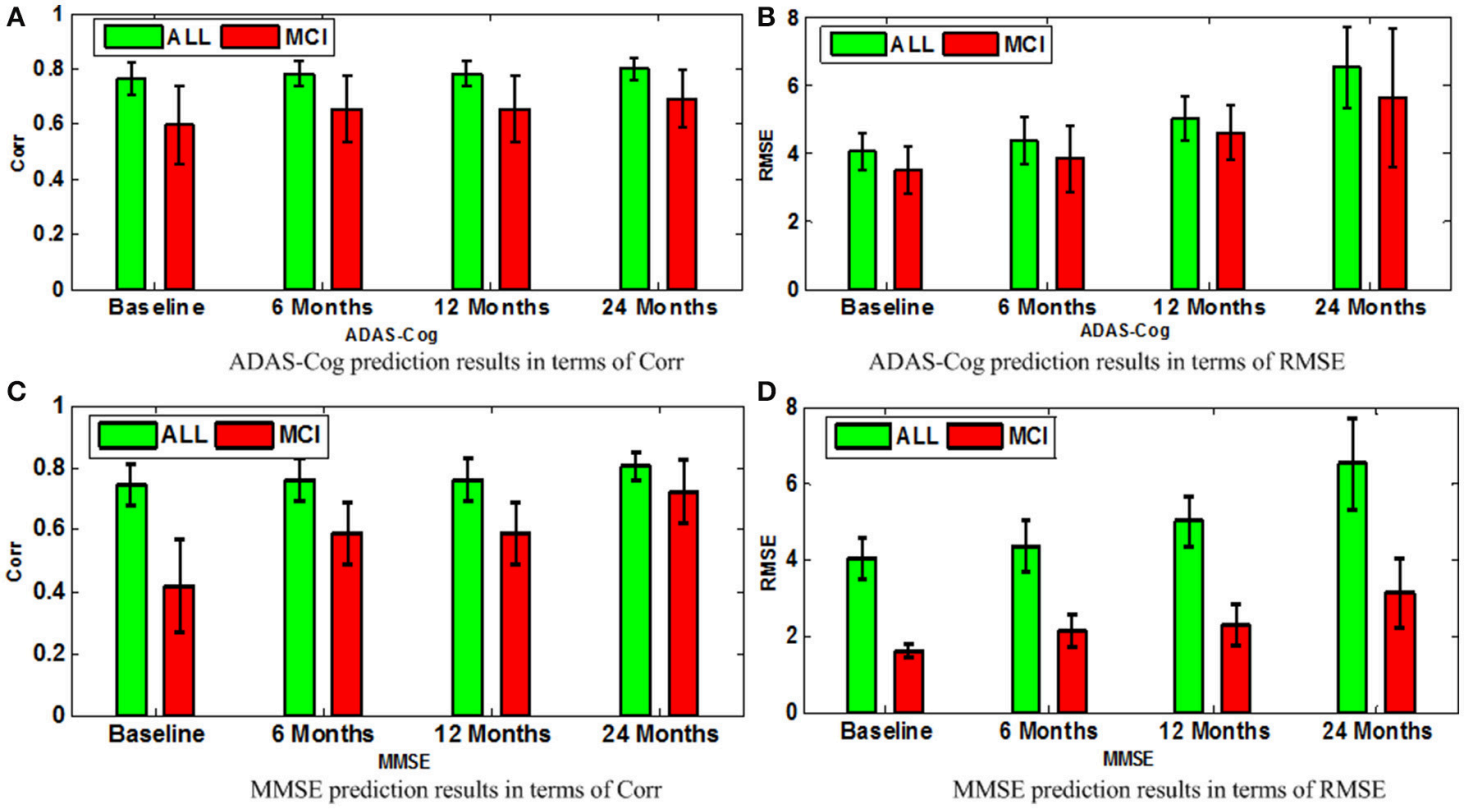
**Comparison of ADAS-Cog and MMSE prediction models in terms of both Corr and RMSE for all subjects including AD, MCI, and NC (ALL) and only MCI patients (MCI); (A)** ADAS-Cog prediction results in terms of Corr **(B)** ADAS-Cog prediction results in terms of RMSE **(C)** MMSE prediction.

Next, we compare different sub-groups containing all subjects including AD, MCI, NC (ALL), and only MCI patients (MCI). Figure 9 presents the detailed comparison results of the different sub-groups for ADAS-Cog and MMSE prediction. Since our method is jointly learned, better results are observed from the ALL group. This group provides the opportunity for the proposed algorithm to discover multi-relationship information using multi-task learning. Table 3 shows the detailed comparison of the state-of-the-art methods. The proposed method with multi-relationship information obtains remarkable results, especially for the prediction of later time points for both ADAS-Cog and MMSE, which are consistent with the findings in the previous publications as well (Zhang et al., 2012; Zhou et al., 2013).

**Table 3:** **Algorithm comparisons of the proposed method with the related works for ADAS-Cog and MMSE prediction**.

**Method**	**Target**	**Subject**	**Feature**	**Result (Corr)**
Duchesne et al., 2009	M12 MMSE	75 NC, 49 MCI, 75 AD	Baseline MRI, age, gender, years of education	MMSE: 0.31 (*p* = 0.03)
Stonnington et al., 2010	Baseline ADAS-Cog and MMSE	Set 1:73 AD, 91 NC Set 2: (ADNI) 113 AD, 351 MCI, 122 NC	BaselineMRI, CSF	MMSE Set1: 0.7 (*p* < 10e-5) Set 2: 0.48 (*p* < 10e-5) ADAS-Cog Set 2: 0.57 (*p* < 10e-5)
Zhang et al., 2012	Baseline,M06,M12,M24 ADAS-Cog, MMSE	ADNI: 91 AD, 202 MCI, 152 NC	Baseline,M06, M12, M24 MRI	Average MMSE: 0.613 (*p* < 10e-5) Average ADAS-Cog: 0.639 (*p* < 10e-5)
Proposed	Baseline, M06, M12, M24 ADAS-Cog, MMSE	ADNI: 91 AD, 202 MCI, 152 NC	Baseline MRI	Average MMSE: 0.7538 (*p* < 10e-5) Average ADAS-Cog: 0.7875 (*p* < 10e-5)

*AD, MCI, and NC denote Alzheimer's disease patients, mild cognitive impairment patients, and normal controls, respectively*.

### Top selected brain regions

**Figure 10:**
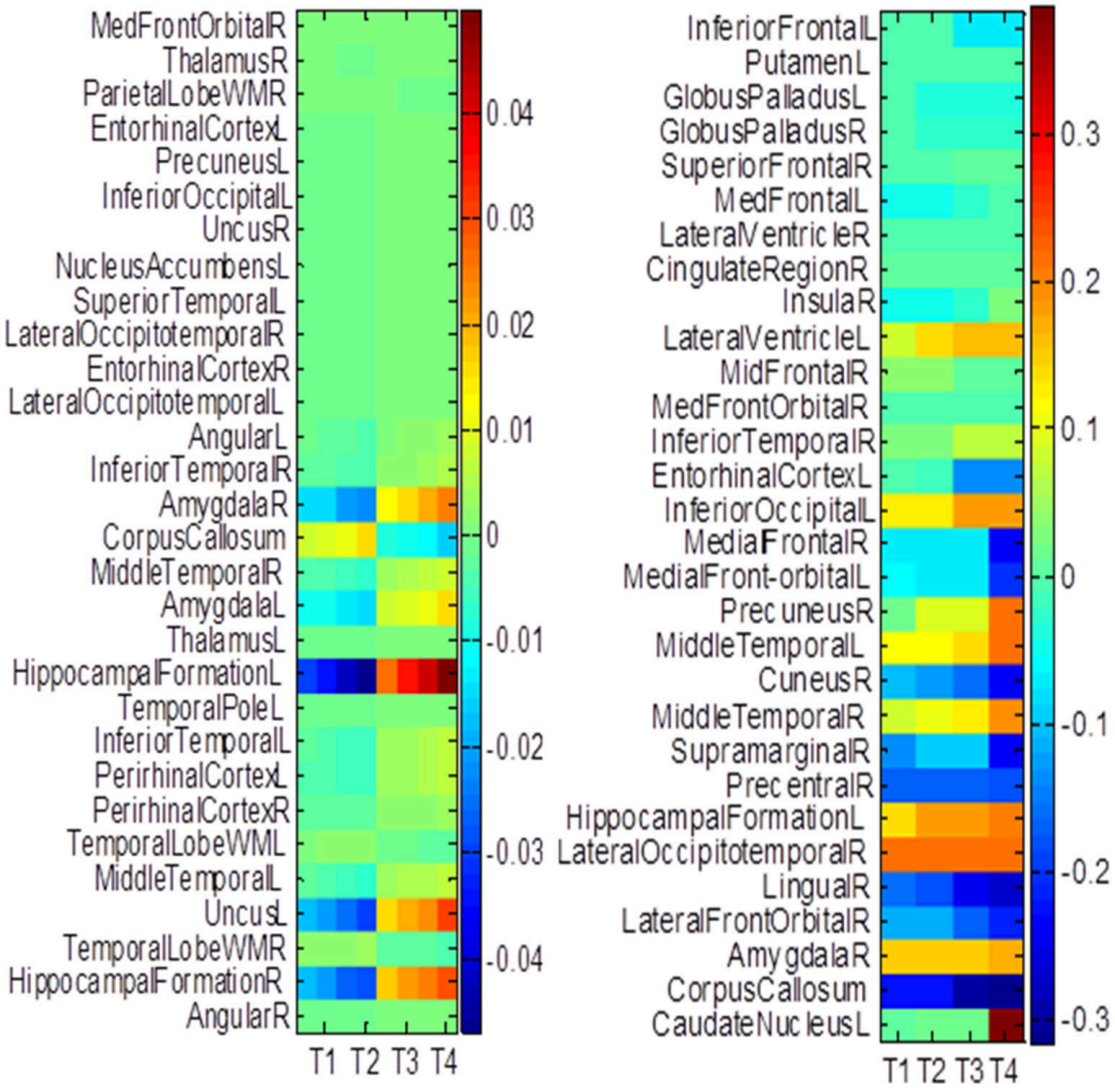
**The most predictive regions identified by (A)** Proposed and **(B)** cFSGL method (Zhou et al., 2013). Note that the proposed method learns ADAS-Cog/MMSE jointly, while cFSGL learns them separately.

The hippocampus plays an important role in identifying brain conditions through AD modeling and measuring the cognitive outcomes such as ADAS-Cog and MMSE. By extracting features from the associated brain regions, the multi-task mechanism accounts for the hippocampus region's sensitivity in cognitive score prediction via MRI baseline data. MCI is characterized by the temporal lobe neocortical regions during dementia decline. Therefore, it is of great significance to find biomarkers for the diagnosis of AD. In this section, we investigate multiple regression variables and their relationships with brain variables such as ADAS-Cog and MMSE. Based on the cross validation, the top predictive regions were selected in terms of the frequency of feature appearance. Figure 10 shows the top 30 most predictive brain regions with the highest weights for ADAS-Cog and MMSE predictions of our proposed and cFSGL methods, where darker colors denote larger weights and vice versa. The most predictive brain regions, namely, the hippocampus and amygdala, are commonly selected for regression tasks. Most of the commonly selected top regions such as hippocampal formation, amygdala, and uncus regions proved to be sensitive and provides AD biomarkers in many studies (Zhang et al., 2012; Zhou et al., 2013).

Biomarkers from different time-points are consistently identified, and important MRI patterns are localized, which suggest that the MRI biomarkers are able to predict the ADAS-Cog and MMSE results effectively. The distinctive and important biomarkers selected in our study included the hippocampal formation, amygdala, middle temporal lobe, uncus, and corpus callosum. The hippocampus is the most important region affecting AD as it possesses significant structural lesions (Convit et al., 2000; Laakso et al., 2000; Wolf et al., 2003; Del Sole et al., 2008; Knafo et al., 2009; Derflinger et al., 2011; Poulin et al., 2011; Ota et al., 2014). Both cFSGL and proposed method selected the important biomarkers for AD diagnosis, while our proposed method identifies more stable and related features for the clinical score measurement. Moreover, the distinctive information selected by the proposed method is more promising for AD modeling than the cFSGL method, due to the joint learning adopted in a multi-task framework, and weights for both ADAS-Cog and MMSE are considered jointly, rather than separately. The common and shared high-level information of ADAS-Cog and MMSE scores can be further explored for disease progression modeling. As a result, the most predictive brain regions such as the hippocampus, amygdala, and temporal patterns are commonly selected in our regression models. The identified brain regions are consistent across multiple time points. The weights for ADAS-Cog and MMSE have similar patterns as well. Moreover, our proposed method confirms that the hippocampus and amygdala are highly important for studies on AD.

**Figure 11:**
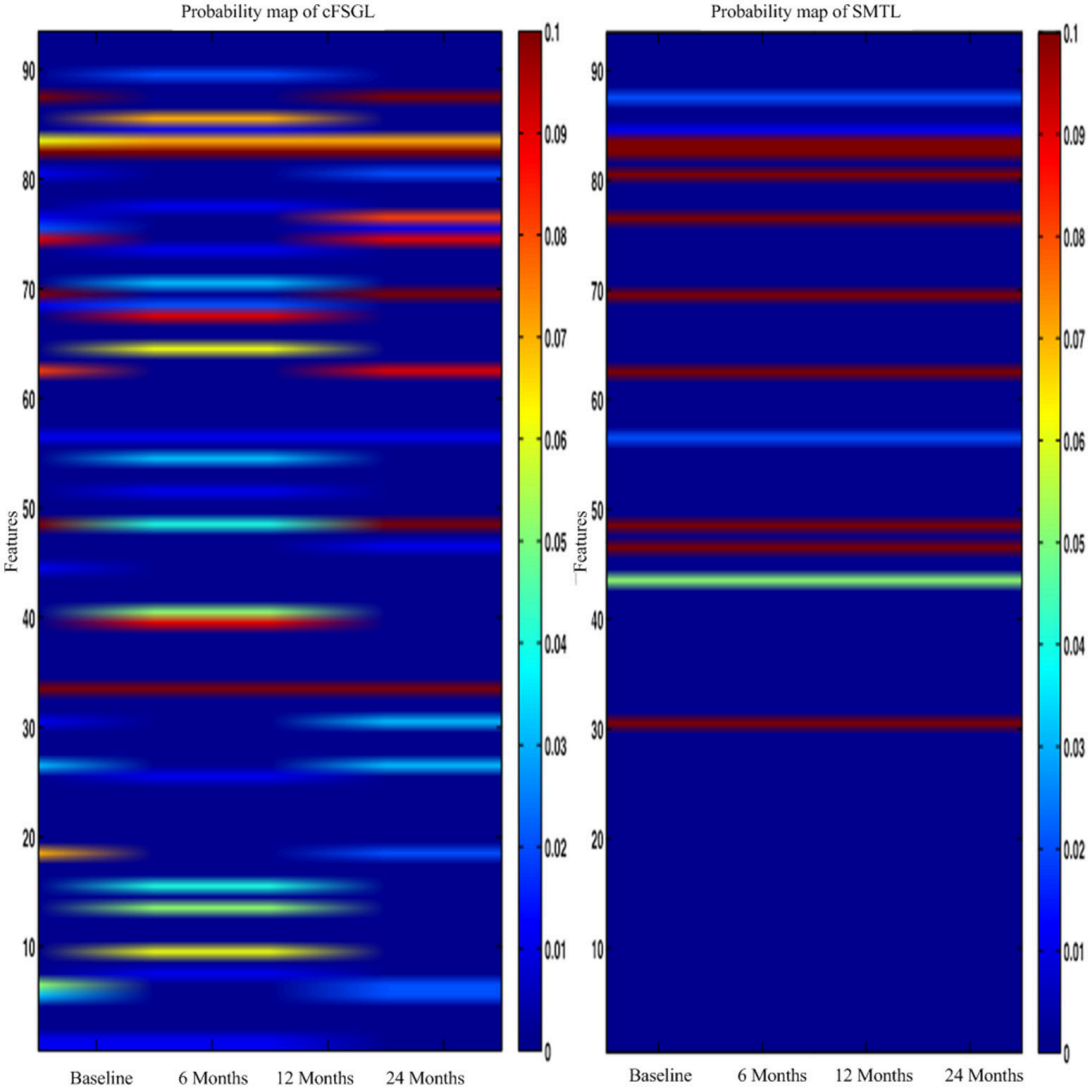
**The probability feature map for predicting the scores**.

In general, higher weights lead to the selection of better features, namely, large weight values correspond to the effectiveness of features to characterize the brain atrophy through quantitative measurements. The experiments on progression from MCI to AD produce similar results from predicting AD and NC in the training steps. ADAS-Cog and MMSE predictions share common effective information. Other feature selection methods such as cFSGL demonstrate different patterns. As shown in the stable feature selection results, our method generally obtains more stable features for predicting both ADAS-Cog and MMSE scores at multiple time points. In general, our method outperforms the competing methods, which is also demonstrated in the probability map shown in Figure 11. The probability map further confirms that the hippocampus and amygdala are highly correlated with AD. The features extracted from these regions have high predictive power for the ADAS-Cog and MMSE prediction.

**Figure 12:**
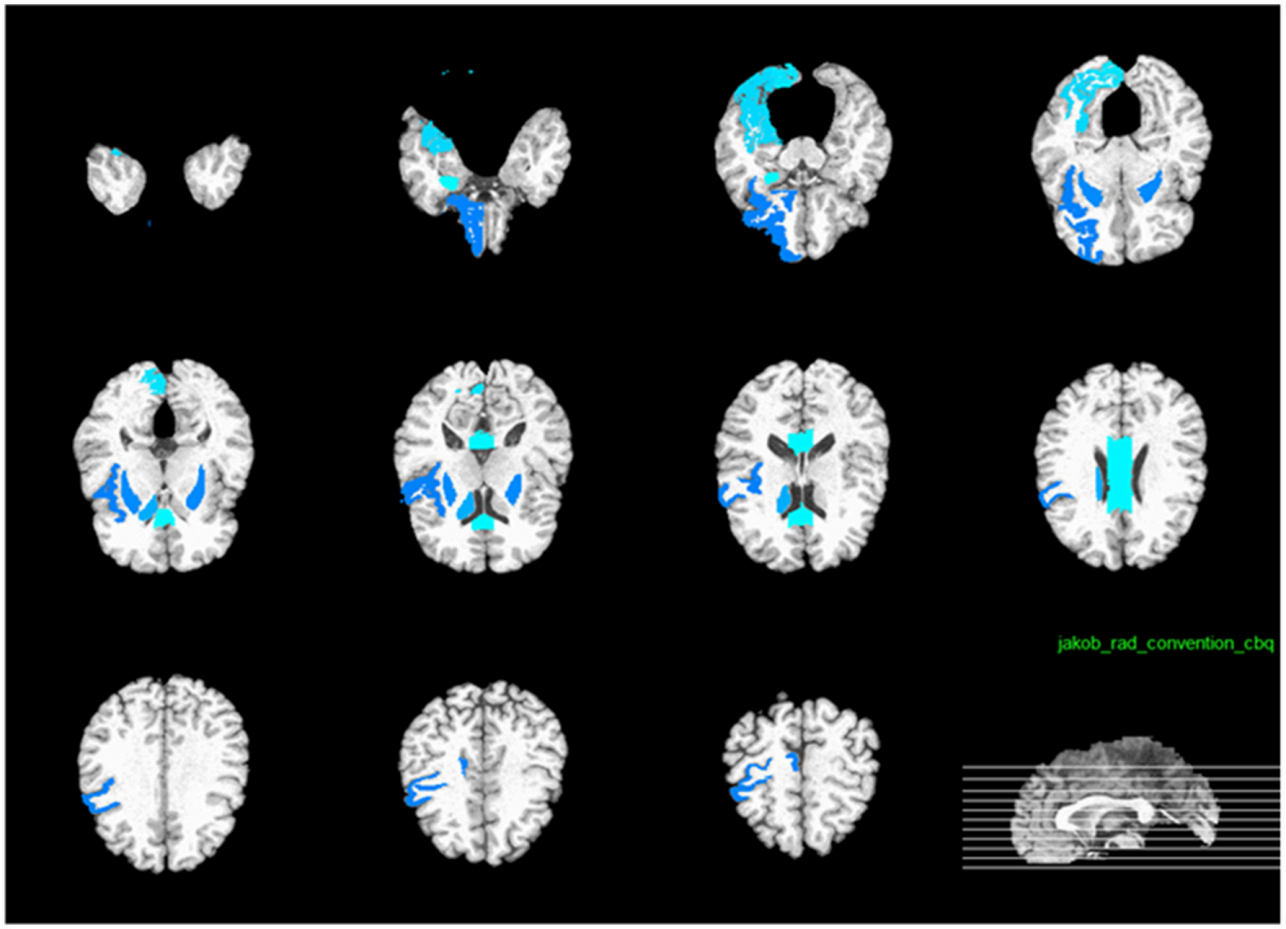
**Top 10 most predictive brain regions for predicting the cognitive measurements**.

As illustrated in Figure 12, the most effective features for predicting ADAS-Cog and MMSE across all time points are, namely, angular R, right hippocampal formation, right temporal lobe WM, left uncus, left middle temporal, right periirhinal cortex, left periirhinal cortex, left inferior temporal, and left temporal. Our findings are consistent with many previous studies which determined the amygdala, hippocampal formation, angular, and uncus are the most predictive biomarkers for AD characterization (Convit et al., 2000; Laakso et al., 2000; Wolf et al., 2003; Del Sole et al., 2008; Knafo et al., 2009; Derflinger et al., 2011; Poulin et al., 2011; Ota et al., 2014).

## Discussions and concluding remarks

These relationships could provide inherent high-level information on AD progression, therefore modeling and utilizing such relationship information can enhance the learning performance of AD progression prediction. Although there are a myriad of progression models available, such as regression and survival model (Wang et al., 2010), we used a popular SVR model due to its desirable properties. The accurate prediction of ADAS-Cog and MMSE scores is essential in modeling disease progression of AD, and to assist clinical assessment. It is shown that clinical scores are highly correlated with ventricle change, shape, hippocampal, and gray matter volume loss. Because the medial temporal lobe and brain atrophy pattern are highly related to MMSE, the correlation should be utilized for clinical score prediction. Apart from feature selection, clinical model and statistical analysis extract diagnostic information and learn large-scale medical imaging based features. The advantages of our models include discrete timestamps at irregular intervals for modeling the disease progression of AD, and the greater interpretability of our model due to the improvement in the selection of distinct features.

Even though promising prediction results were obtained in our study, there are still some limitations in the proposed feature selection method. First, only a single modality is used. If multiple modalities were available (such as PET, DTI, APOE, and CSF), the predictive power could be possibly enhanced. Second, all subjects should have complete feature values with no missing data. This would be challenging to achieve with larger experiments and more subjects. Future research can be conducted to investigate the issues associated with missing data. Third, we only investigate the ADAS-Cog and MMSE predictions. It is possible to extend this method to predict the sub-scores of clinical tests and other regression variables, such as the clinical dementia rating scale sum of boxes (CDR-SOB) and the auditory verbal learning test (AVLT) for cognitive measurement.

A problem that is often referred to as the curse of dimensionality, where the large size (i.e., large number of dimensions) of features for modeling AD progression makes it difficult to perform various numerical analyses on the data. This problem led to increased difficulty to draw consistent conclusions from the dataset. The feature selection method addressed this problem by leveraging the sparse learning technique to model the AD progressive and predict AD progression using the temporal priors and hippocampus. Apart from feature selection, the clinical model and statistical analysis extracted diagnostic information and learned large-scale medical imaging based on features. By merging fused multi-task learning together with the temporal smoothing of the parametric hippocampus surface, clinical scores, and subjects, promising prediction results for future ADAS-Cog and MMSE prediction were obtained.

The main challenge is to identify task-specific features and significant biomarkers to model AD, as well as locating a common set of features using the learned model. Previous works showed that performance decreases with a smaller training set (Stonnington et al., 2010), but the trend and relative performance remain comparable. An interesting direction is to add constraints to find similar parametric surfacesto determine more similar and smoother weights, and hence better and more consistent results can be obtained. This work illustrated that the extraction of the volumetric information from parametric surface could aid in the prediction of AD progression, which could be also extended to fMRI studies. Because the dimensionality problem still exists when the number of voxel and vertex points increases, feature selection is generally a plausible approach to leverage prior information and explore sparsity and smoothness as well. Sparse learning is a powerful tool to identify useful features and reduce feature dimension. Although there are a number of feature selection algorithms available, the features from these algorithms often lack biological meanings and reasonable interpretations. A method that is capable of locating desirable features with reasonable feature dimension is highly desirable.

Directions for future work include understanding the behavior of weights across the parametric surface space and time. Previous works shown that stability selection (Zhou et al., 2013) may be a good fit for analyzing the feature weights on the model. Future works including the stability analysis of weights which might provide more information on the relationship between the deformation of hippocampal subfields and other clinical indicators, such as AVLT during AD progression. The longitudinal information on brain structure is highly correlated to disease progression. Hippocampal atrophic rates and ventricular changes are assessed statistically with the surface change. The resulting maps are sensitive to longitudinal changes in brain structure as the disease progresses. Additional maps to localize atrophic change regions are linked to cognitive decline. Additional maps for hippocampal atrophy and clinical deterioration are also helpful for understanding AD progression. These quantitative, dynamic visualizations of hippocampal atrophy and ventricular expansion rates in aging and AD may provide a promising measure to track AD progression in drug trials. Furthermore, it would be interesting to investigate the feasibility of extending our joint learning method to model and predict other diseases, such as Parkinson's disease or Autism disorder.

In this paper, we proposed a novel longitudinal prediction model which incorporates multiple relation information of data in a unified objective function. We applied our proposed model for AD progression prediction at multiple future time points, using the baseline data only. Specifically, we developed a novel multi-task sparse feature selection model by considering the relationships between features, subjects, and clinical scores. The feature selection procedure selects the most relevant features for the task of clinical scores prediction at multiple future time points, followed by the use of regression models for predictions. Our experimental results of the proposed method based on the ADNI database demonstrated promising results in estimating the clinical cognitive scores at multiple future time points.

## Author contributions

BL, FJ, SC, DN, and TW designed the experiments; BL and DN performed the data analysis and experiments; BL wrote the main manuscript text; SC and TW provided scientific interpretation; BL, FJ, SC, DN, and TW reviewed the manuscript, provided final approval of the version to be published and ensured the accuracy and integrity of the work.

### Conflict of interest statement

The authors declare that the research was conducted in the absence of any commercial or financial relationships that could be construed as a potential conflict of interest.
